# Global landscape analysis of antibody-drug conjugate clinical trials for prostatic neoplasms: current status, opportunities and challenges

**DOI:** 10.3389/fimmu.2026.1774353

**Published:** 2026-05-14

**Authors:** Shuyu Ren, Fangfang Pan, Jie Jin

**Affiliations:** Rehabilitation Department, Second Affiliated Hospital, Zhejiang University School of Medicine, Linping Campus, Hangzhou, China

**Keywords:** antibody drug conjugate (ADC), B7-H3, clinical trial landscape analysis, DS-7300, prostatic neoplasms, protease cleavable linker, topoisomelase-1 inhibitor payloads, tumour heterogeneity

## Abstract

Antibody-drug conjugates (ADCs) offer selective delivery of potent cytotoxic payloads to tumour-associated antigens and are therefore attractive candidates for biomarker-informed treatment development. However, antigen heterogeneity, payload resistance, linker stability, and on-target/off-tumour toxicities continue to complicate clinical translation. Several next-generation ADC programmes, including PSMA- and B7-H3-directed agents such as MEDI3726 and DS-7300, have reported early antitumour activity signals. At the same time, the global development landscape remains fragmented across targets, payload classes, endpoints, and sponsor types. This study therefore aimed to map the contemporary landscape of ADC clinical trials for prostatic neoplasms, clarify current development patterns, identify translational opportunities and gaps, and provide an integrated overview to support future clinical development.

## Introduction

The global burden of prostatic neoplasms is projected to surge from 1.4 million new cases in 2020 to 2.9 million by 2040, with deaths potentially rising by approximately 85% (1). In current clinical practice, treatment pathways for metastatic hormone-sensitive prostate cancer (mHSPC) and metastatic castration-resistant prostate cancer (mCRPC) remain dominated by androgen-deprivation-based intensification, androgen receptor pathway inhibitors (ARPIs), taxanes, PARP-based precision therapy in selected patients, and radioligand approaches in appropriate settings (2–5). Yet even landmark therapies like ^177^Lu-PSMA-617 extend median overall survival only from 11.3 months to 15.3 months, highlighting persistent resistance despite multi-line treatment (2).

## Methods

This study used the Trialtrove database (https://clinicalintelligence.citeline.com/), a curated commercial subscription database of global clinical trial intelligence. The database is not publicly open-access; rather, it compiles and curates trial information from multiple sources, including major public registries, company disclosures, medical meeting materials, and other public-domain trial information sources. The Trialtrove query was run on 12 October 2025 using the indexed filters “Drug: Antibody-drug conjugate” and “Patient population: Prostate tumour”. Eligible records were interventional clinical trials involving ADCs in prostatic neoplasms. We excluded observational studies, antibody-radionuclide conjugate studies, non-ADC drug trials, and enrolment-only or sub-arm records within multi-arm/master-protocol studies if inclusion would duplicate the underlying ADC platform. After screening and deduplication, 118 eligible clinical trials were included.

Two independent researchers cross-checked extracted variables to improve data quality and reduce classification error. The extracted fields included trial phase, recruitment status, start year, target, linker type, payload class, patient stage/line of therapy, primary endpoints, sponsor category, and geographic distribution. When a trial was linked to multiple countries, disease stages, or treatment settings, category counts could exceed the number of unique studies. This study was designed as a landscape analysis rather than a comparative effectiveness review; therefore, we did not perform a formal trial-level risk-of-bias assessment or meta-analysis. Furthermore, this research adhered to the normative requirements stipulated in the TITAN Guidelines 2025 (6).

## Results

**Figure 1:**
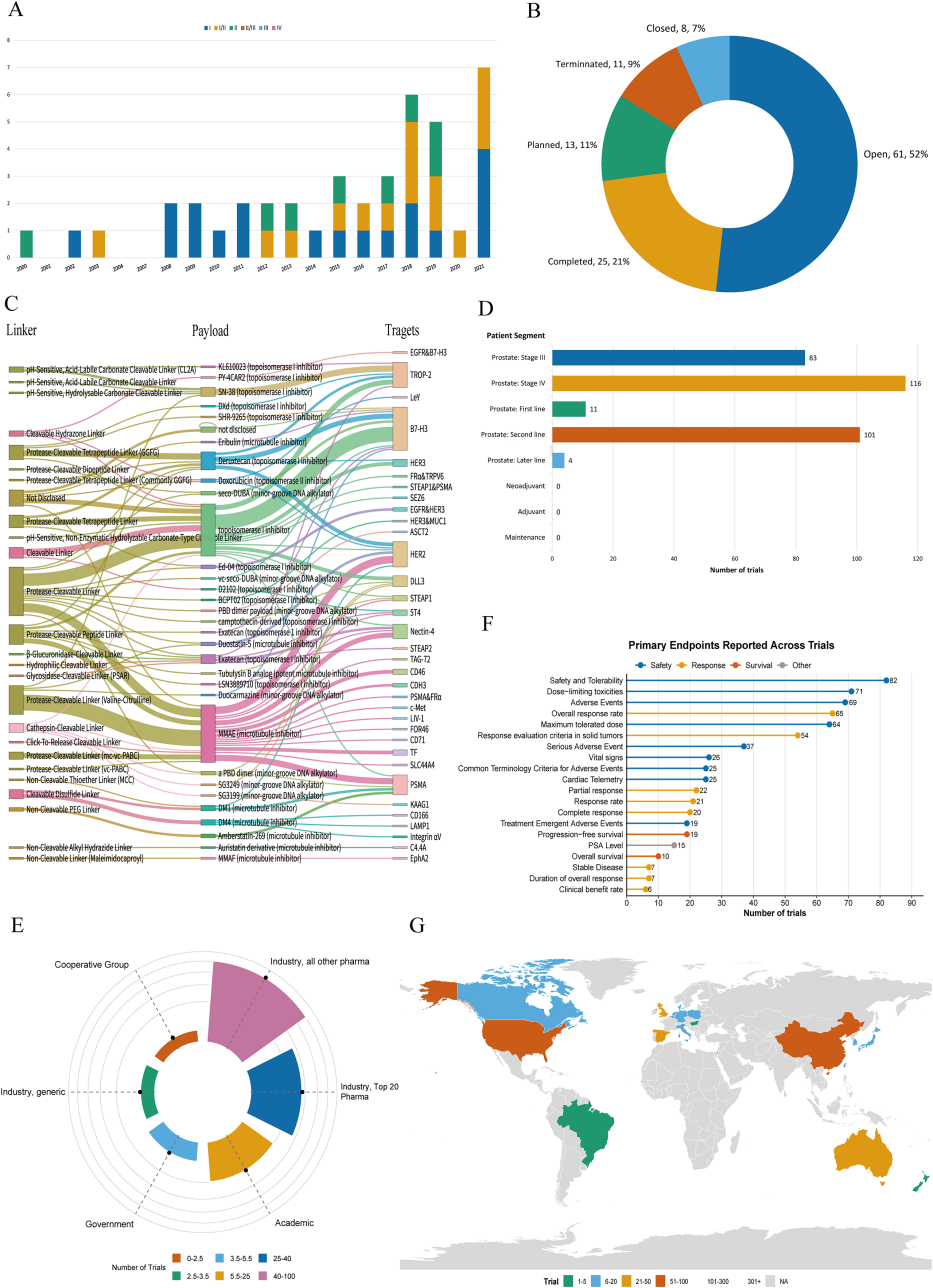
Global landscape of antibody-drug conjugate clinical trials for prostatic neoplasms. Data are based on 118 eligible antibody-drug conjugate (ADC) clinical trials identified from the Trialtrove database. **(A)** Annual distribution of ADC clinical trials by start year and clinical phase. **(B)** Distribution of trial status. **(C)** Sankey diagram showing the relationships among linker type, payload, and molecular target. **(D)** Distribution of enrolled patient segments by disease stage and treatment line or setting. **(E)** Distribution of sponsor categories. **(F)** Primary endpoints reported across trials, grouped as safety, response, survival, and other endpoints. **(G)** Country-level geographic distribution of trial locations. Category counts may exceed the number of unique trials because individual trials could be assigned to multiple countries, patient segments, treatment settings, targets, payloads, or endpoint categories.

ADC clinical trials for prostatic neoplasms have entered a phase of rapid expansion, with 66 studies initiated during the peak three-year period from 2023 to 2025. However, early exploratory trials (Phase I/I-II) continue to dominate the field, accounting for 90 of 118 studies (76.3%), indicating that hypothesis validation and early dose exploration remain the principal focus. (**Figure 1A**) With respect to development status, 61 trials (51.7%) were ongoing and 25 (21.2%) had been completed, together representing nearly three-quarters of the pipeline, while 13 trials (11.0%) were planned and 19 (16.1%) had been terminated or closed. (**Figure 1B**) Protease-cleavable linkers accounted for 68.1% of platforms, with Val-Cit as the most common individual linker type (13.5%). Topoisomerase I (TOP1)-based payloads represented 52.1% of trials, compared with 38.7% for microtubule inhibitors and 7.6% for DNA-alkylating agents, indicating a clear current preference for TOP1-oriented payload design. Target selection had diversified to 35 antigens. Beyond PSMA (9.2%), frequently represented targets included B7-H3 (20.2%), TROP2 (11.8%), and HER2 (11.8%). This diversification suggests an active effort to address tumour heterogeneity through broader antigen exploration. (**Figure 1C**) The enrolled populations were concentrated in advanced and treatment-experienced settings. Stage IV patient categories (n=116) outnumbered Stage III categories (n=83), while second-line settings (n=101) exceeded first-line settings (n=11) and hormone-resistant populations remained prominent (n=80). This reflects the development strategy within this field. (**Figure 1D**) The sponsorship structure was dominated by small and medium-sized biopharmaceutical companies (64/118, 54.2%), followed by the top 20 pharmaceutical companies (28/118, 23.7%), collaborative or academic/government-associated funding (23/118, 19.5%), and generic manufacturers (3/118, 2.5%). (**Figure 1E**) Primary endpoints were dominated by safety and dose-finding metrics, including safety/tolerability (n=82), dose-limiting toxicity (n=71), adverse events (n=69), and maximum tolerated dose (n=64). Efficacy readouts were more limited and centred mainly on objective response rate and RECIST-based assessment (n=65 and n=54, respectively), whereas time-to-event endpoints were sparse, with progression-free survival (PFS) reported in only 19 trials and overall survival (OS) in 10 trials. The limited representation of PFS (n=19) and OS (n=10) underscores the current lack of mature time-to-event evidence. (**Figure 1F**) Geographically, ADC trials for prostatic neoplasms showed a North America-Asia concentration pattern with broad but less intensive European participation. The United States contributed 90 of 377 country-level trial entries (23.9%) and China contributed 54 (14.3%), together accounting for 38.2% of total geographic entries. The top five countries—the United States, China, Australia, Spain, and the United Kingdom—accounted for 226 of 377 entries (60.0%), indicating marked concentration of development resources. Australia (29 entries), Spain (28 entries), and the United Kingdom (25 entries) also showed substantial activity, whereas Latin America and Central/Eastern Europe were sparsely represented. (**Figure 1G**).

## Discussion

This study was designed to map the development landscape rather than to rank individual ADC platforms by comparative efficacy or safety. Within this framework, ADCs are most plausibly positioned as investigational, biomarker-guided options for patients with resistant or heavily pretreated disease, particularly after prior ARPI exposure, rather than as immediate replacements for existing backbones. At present, standard treatment sequences in mHSPC and mCRPC remain anchored by ARPI-based intensification, taxanes, selected PARP inhibitor strategies, and radioligand therapy in appropriate settings (2–5). Because ADC cytotoxicity is driven by target engagement and payload delivery rather than direct androgen-receptor inhibition, these agents might partly bypass some mechanisms of ARPI resistance. However, current evidence is not sufficient to conclude that ADCs reverse established ARPI resistance, and this question requires prospective clinical testing.

Clinical efficacy data remain early and exploratory rather than practice-changing. In an earlier phase I study, the PSMA-targeted ADC MEDI3726 reported preliminary antitumour activity in patients with mCRPC after failure of abiraterone or enzalutamide (7). In the phase I/II subgroup analysis of DS-7300 in mCRPC, RECIST responses were observed across dose levels, including six partial responses (four confirmed), with grade ≥3 treatment-emergent adverse events in 34.5% of patients; the most common severe event was anaemia, and no ILD/pneumonitis was reported (8). These data support biological activity and tolerability, but they remain preliminary and were generated in a small, heavily pretreated population. In the randomised phase II TAMARACK study, vobramitamab duocarmazine achieved 6-month radiographic progression-free survival rates of 69-70%, with confirmed ORRs of 20.0% and 40.6% in the 2.0 mg/kg and 2.7 mg/kg cohorts, respectively, among RECIST-evaluable patients (9). However, grade ≥3 treatment-emergent adverse events remained frequent (62.8-65.6%), dose reductions were common, and fatal treatment-related events including pneumonitis were reported (9). These findings indicate that antitumour activity can be observed in selected ADC programmes, but they also underline that dose-toxicity balance remains a central challenge for clinical development.

Biological context also supports continued target exploration. B7-H3 is broadly expressed in aggressive prostate cancer phenotypes and has shown translational relevance in RB1-deficient/neuroendocrine prostate cancer biology and preclinical biomarker studies (10, 11). At the same time, PSMA expression is heterogeneous and dynamic, and recent tissue-based work has highlighted the need to identify alternative actionable antigens in PSMA-low or PSMA-negative disease (12). Together, these observations reinforce the need for biomarker-guided ADC development rather than one-size-fits-all target selection.

The endpoint structure observed in this study has direct translational implications. The dominance of safety, DLT, AE, and MTD endpoints is appropriate for an early-phase field, but the relative scarcity of time-to-event endpoints limits interpretation of durable clinical benefit and constrains cross-platform comparison. Without more mature PFS, OS, and patient-centred outcome data, it remains difficult to determine how ADCs should be sequenced against guideline-endorsed therapies or how broadly they could support future regulatory positioning. This pattern also reinforces the need to move from empiric dose escalation toward earlier randomised dose-optimisation strategies aligned with contemporary ADC regulatory expectations (13, 14).

The dominance of TOP1 payloads likely reflects several practical advantages in solid tumours: compatibility with cleavable linkers, the feasibility of higher drug-to-antibody ratios, membrane-permeable payload catabolites, and potential bystander killing in biologically heterogeneous tumours (15, 16). These characteristics are especially attractive in prostate cancer, where intratumoural and intermetastatic heterogeneity may limit the uniformity of target expression. Future development should prioritise rational combination strategies rather than isolated escalation of single agents. ARPIs remain a clinical backbone in both hormone-sensitive and castration-resistant settings, making them logical partners or anchoring comparators for ADC development. Combination concepts involving PARP inhibitors, radiotherapy, or biomarker-guided sequencing may also be relevant, particularly where DNA damage response biology, oligometastatic control, or antigen heterogeneity create a sound mechanistic rationale (11, 4, 5).

Clinically relevant toxicity management will be equally important for adoption. Treatment-related interstitial lung disease/pneumonitis deserves proactive surveillance, especially for deruxtecan-like payload platforms (9, 13). In advanced prostate cancer populations with frequent bone involvement, skeletal-related events, marrow reserve, and supportive bone-targeted care must also be considered during trial design and real-world implementation (17). Sequential biomarker monitoring, imaging integration, and adaptive trial platforms may help refine both patient selection and toxicity management (18).

This study has several limitations. First, it is a descriptive clinical-trial landscape analysis and does not provide a head-to-head ranking of ADC programmes by comparative efficacy, safety, or clinical maturity. Second, the dataset was derived exclusively from Trialtrove, a subscription-based curated database; although it integrates multiple global sources, region-specific studies, unregistered studies, or recently updated records may have been missed. Third, because the aim was to characterise development patterns rather than evaluate treatment effects, we did not perform a formal trial-level quality or risk-of-bias assessment. These limitations should be considered when interpreting the present findings.

## Conclusion

The accelerated development of ADCs for prostatic neoplasms appears to be driven by target diversification, growing maturity in TOP1-based medicinal chemistry, and increasing emphasis on biomarker-informed development. Nonetheless, the field remains predominantly early phase, and current efficacy signals should be interpreted as promising but preliminary. The next stage of progress will likely depend on three linked transitions: from empiric dose escalation to systematic dose optimisation, from passive safety reporting to proactive toxicity management, and from static biomarker use to adaptive patient-selection frameworks that integrate pathology, imaging, and molecular profiling. Achieving these transitions within collaborative, biomarker-aware clinical development programmes will be essential for translating ADCs into meaningful treatment options for prostate cancer.

## Data Availability

The original contributions presented in the study are included in the article/**Supplementary Material**. Further inquiries can be directed to the corresponding author.

## References

[1] Che J Liu Y Liu Y Song J Cui H Feng D . The application of emerging immunotherapy in the treatment of prostate cancer: progress, dilemma and promise. Front Immunol. (2025) 16:1544882. doi: 10.3389/fimmu.2025.1544882. PMID: 40145100 PMC11937122

[2] Giraudet AL Kryza D Hofman M Moreau A Fizazi K Flechon A . PSMA targeting in metastatic castration-resistant prostate cancer: where are we and where are we going? Ther Adv Med Oncol. (2021) 13:17588359211053898. doi: 10.1177/17588359211053898. PMID: 34721674 PMC8554551

[3] Garje R Bin Riaz I Naqvi SAA Rumble RB Taplin ME Kungel TM . Systemic therapy in patients with metastatic castration-resistant prostate cancer: ASCO guideline update. J Clin Oncol. (2025) 43:2311–34. doi: 10.1200/JCO-25-00007. PMID: 40315400

[4] Lee CH Kim S Ku JY Kim KH Kang BJ Goh HJ . Comparative effectiveness of androgen receptor pathway inhibitor treatment intensification for metastatic hormone-sensitive prostate cancer in real-world patients. Investig Clin Urol. (2025) 66:516–25. doi: 10.4111/icu.20250313. PMID: 41184145 PMC12599407

[5] Encarnación JA Morillo Macías V De la Fuente Muñoz I Soria VD Fernández Fornos L Antequera MA . Apalutamide and stereotactic body radiotherapy in metastatic hormone-sensitive prostate cancer: multicenter real-world study. Cancers (Basel). (2025) 17:2216. doi: 10.3390/cancers17132216. PMID: 40647514 PMC12249003

[6] Agha RA Mathew G Rashid R Kerwan A Al-Jabir A Sohrabi C . Transparency in the reporting of artificial intelligence – the TITAN guideline. Premier J Sci. (2025) 10:100082. doi: 10.70389/PJS.100082

[7] de Bono JS Fleming MT Wang JS Cathomas R Miralles MS Bothos J . Phase I study of MEDI3726: a prostate-specific membrane antigen-targeted antibody-drug conjugate in patients with mCRPC after failure of abiraterone or enzalutamide. Clin Cancer Res. (2021) 27:3602–9. doi: 10.1158/1078-0432.CCR-20-4528. PMID: 33795255

[8] Patel MR Johnson ML Falchook GS Doi T Friedman CF Piha-Paul SA . DS-7300 (B7-H3 DXd-ADC) in patients with metastatic castration-resistant prostate cancer: a subgroup analysis of a phase 1/2 multicenter study. J Clin Oncol. (2022) 40:87. doi: 10.1200/JCO.2022.40.6_suppl.087. PMID: 41909186

[9] MacroGenics . MacroGenics Announces Updated Efficacy & Safety Data from TAMARACK Phase 2 Study of Vobramitamab Duocarmazine in mCRPC Patients at ESMO Congress 2024. Rockville, MD: MacroGenics, Inc. (2024).

[10] Yamada Y Venkadakrishnan VB Mizuno K Bakht M Ku SY Garcia MM . Targeting DNA methylation and B7-H3 in RB1-deficient and neuroendocrine prostate cancer. Sci Transl Med. (2023) 15:eadf6732. doi: 10.1126/scitranslmed.adf6732. PMID: 37967200 PMC10954288

[11] Agarwal S Fang L McGowen K Yin J Bowman J Ku AT . Tumor-derived biomarkers predict efficacy of B7H3 antibody-drug conjugate treatment in metastatic prostate cancer models. J Clin Invest. (2023) 133:e162148. doi: 10.1172/JCI162148. PMID: 37725435 PMC10645377

[12] Mulati Y Shen Q Tian Y Chen Y Yao K Yu W . Characterizing PSMA heterogeneity in prostate cancer and identifying clinically actionable tumor associated antigens in PSMA-negative cases. Sci Rep. (2025) 15:23902. doi: 10.1038/s41598-025-06393-z. PMID: 40615466 PMC12227638

[13] U.S. Food and Drug Administration . Clinical pharmacology considerations for antibody-drug conjugates: guidance for industry. Silver Spring, MD: U.S. Food and Drug (2024).

[14] Fourie Zirkelbach J Shah M Vallejo J Cheng J Ayyoub A Liu J . Improving dose-optimization processes used in oncology drug development to minimize toxicity and maximize benefit to patients. J Clin Oncol. (2022) 40:3489–500. doi: 10.1200/JCO.22.00371. PMID: 36095296

[15] Kondrashov A Sapkota S Sharma A Riano I Kurzrock R Adashek JJ . Antibody-drug conjugates in solid tumor oncology: an effectiveness payday with a targeted payload. Pharmaceutics. (2023) 15:2160. doi: 10.3390/pharmaceutics15082160. PMID: 37631374 PMC10459723

[16] Conilh L Sadilkova L Viricel W Dumontet C . Payload diversification: a key step in the development of antibody-drug conjugates. J Hematol Oncol. (2023) 16:3. doi: 10.1186/s13045-022-01397-y. PMID: 36650546 PMC9847035

[17] Mitchell AP Meza AM Panageas KS Lipitz-Snyderman A Farooki A Morris MJ . Real-world use of bone modifying agents in metastatic, castration-resistant prostate cancer. Prostate Cancer Prostatic Dis. (2023) 26:126–32. doi: 10.1038/s41391-022-00573-y. PMID: 35798857 PMC10251421

[18] Hong J Bae S Cavinato L Seifert R Ryhiner M Rominger A . Deciphering the effects of radiopharmaceutical therapy in the tumor microenvironment of prostate cancer: an in-silico exploration with spatial transcriptomics. Theranostics. (2024) 14:7122–39. doi: 10.7150/thno.99516. PMID: 39629134 PMC11610134

